# Opening of the New Wing at Poplar Hospital

**Published:** 1901-06-29

**Authors:** 


### The Institutional Workshop.

### OPENING OF THE NEW WING AT POPLAR HOSPITAL.

Flags and banners waving in the breeze proved that Poplar Hospital was *en fête* on Thursday, 20th inst., when the new medical wing (to be known as the Drapers' Company's Wing) was opened by the Lord Bishop of London. A large tent was put up in the space adjoining the hospital, and the sending out of some hundreds of invitations resulted in a large gathering of friends. The Hon. Sydney Holland was in the chair, and there were also on the platform the Lord Bishop, the Rev. A. N. Campbell (hon. chaplain), the Mayor, the committee of the hospital, the matron (Miss Bland), and others. The Band of the "K" Division of the Metropolitan Police gave a programme of music and accompanied the hymns which formed part of the ceremony.

Mr. Holland said it was a day of triumph, and all concerned had helped to make it so. They were happy to have with them the Bishop, who knew the needs of East London so well; and Dr. Brinfield, their first surgeon, who had admitted the first case, was also on the platform. The reason for extension was the same as for starting this hospital, viz., the miserably inadequate provision for the sick of East London. The West had come to the help of the East, and then came the Drapers' Company, who always took a broad view of their duty. They had offered to help towards the addition of 20 beds; it had been found, however, that it was more economical to build for 30, and the Drapers' Company had given £10,000. £700 was still required; he would not ask for it, but if anybody felt inclined to give it he would be happy to see them in the vestry after the service. The cost of the ceremony would be £100. There were two things he would wish to emphasise: first, young and vigorous men were wanted to work for the hospitals; and secondly, they had determined to open the new wards without letters of admission. He concluded with an eloquent appeal to those who imagined that without letters subscribers did not get their money's worth.

Dr. Dalton, Master of the Drapers' Company, said the members of his court looked upon this matter as business men and as trustees. For every £1 they had to give there were fifteen people asking for it, and they were sorry for the disappointed ones; in this case, however, there were no misgivings, and they believed that the more the East End kept in touch with the City the better for both. There was great deficiency in hospital accommodation in the East End. The London Hospital was doing a great deal, but it was hampered for want of money. The provision of hospitals was no party question, and it did not tend to pauperise the poor. It was a great source of satisfaction to the company to know that patients would have the best attention both day and night; it was a work which they believed had a very humanising influence.

The Bishop of London said in coming to the East End he felt he was at home again, and that was a very great pleasure. It was also a great pleasure to do something for a hospital, and parish priests knew what hospitals meant to the people, especially in the hot days of summer. "We believe," he said, "in the hospitals of East London. Those who try to paint doctors as hard, scientific men have not seen many of them. Those who have worked in East London know the popularity of the Poplar Hospital. I think it is partly due to the chairman, and I confess he quite took me in by the notice pinned up everywhere, that 'money and valuables should be left with the chairman of the hospital.'" They had an excellent and devoted matron and chaplain, and he could not help noticing the extraordinary politeness displayed on the boards outside, one of which requested the drivers to drive slowly, while the other thanked them for doing so. But the main reason for its popularity was that the people knew that when they or their fellows were broken in limb, they received there every attention, and that the hospital was permeated with the true spirit of Christianity. The Drapers' Company deserved the gratitude of the London diocese; he felt very grateful for the work of the past 46 years, and prayed that it might continue. In three weeks' time he would be coming again, to bless the *Discovery*, but to-day they were launching an even more beneficent ship than that. In the name of them all he would say God speed the Poplar Hospital, and declare it open for their good. The hymn "All people that on earth do dwell" having been sung, the Bishop said a prayer for the usefulness of the new wing, and the singing of the National Anthem closed the ceremony.

The addition is of a lofty block of buildings costing about £14,000, situated upon the east side of the surgical ward block.

The funds for the structure have principally been provided by the munificence of the Drapers' Company, who have given £10,000 for this purpose, and the buildings provide the following additional accommodation:—The basement is devoted to the out-patients' department, in which surgical and medical cases are separated, with separate waiting, examining, and dressing rooms for each department, the former out-patients' department being converted into a spacious general waiting room. Upon the ground, first and second floors are the three wards, 41 feet long by 28 feet 6 inches wide, for 10 medical cases each. One is unfurnished owing to lack of funds. They are approached through the old surgical wards, but have in addition their own staircase and outside escape stairs, with ward, scullery, bath-room, sink-room, and other sanitary accommodation in spurs at the east end, and the sisters' room at the west end, and day rooms upon the ground and first floors. Each ward has a wide balcony upon the south front overlooking the docks and river. The floors are laid in ornamental Terrazzo mosaic in panels and borders; the walls and ceilings are plastered; Teale Faience centre ward warm-air stoves are used; the windows are lofty, and there are extract ventilation flues and the usual rounded and hollowed angles throughout. The third floor is devoted to the nursing staff, and includes a nurses' sitting room, 32 feet by 12 feet 6 inches, with large square bay, and seven nurses' bedrooms, besides linen storage and the necessary sanitary accommodation. The sitting-room, which has been pleasantly decorated and furnished, faces the south and south-east, and extensive views over the Thames valley and Kentish Hills are obtained from its windows. The fourth and fifth floors are occupied by servants' bedrooms, and a broad flat for recreation, a portion of which is covered with a shelter roof. The new buildings, like the old, are constructed throughout in a substantial manner, with fireproof floors and stairways, all in the best and most durable materials, and much attention has been paid to the perfection of the sanitary work. No elaboration has been attempted in the elevation; the qualities of light and shade and the effect of magnitude are relied upon to relieve the utilitarian character of the building, whilst the boldly springing gable and the turret roof of the shelter give a picturesque skyline, and make the lofty building a conspicuous and interesting object from all parts of the district. The builders are Messrs. Harris and Wardrop, of Limehouse, who have executed this and the whole of the other additions in connection with the main hospital building.

